# Innovative Non-Thermal Processing Technologies for Shelf Life Extension and Retention of Bioactive Compounds in Liquid Foods: Current Status and Future Prospects

**DOI:** 10.3390/foods14172953

**Published:** 2025-08-25

**Authors:** Muhammad Arslan, Muhammad Zareef, Mubrrah Afzal, Haroon Elrasheid Tahir, Zhihua Li, Halah Aalim, Hamza M. A. Abaker, Xiaobo Zou

**Affiliations:** Agricultural Product Processing and Storage Lab, School of Food and Biological Engineering, Jiangsu University, Zhenjiang 212013, China; zareef@ujs.edu.cn (M.Z.); mubrrahafzal345@gmail.com (M.A.); haroona28@ujs.edu.cn (H.E.T.); lizh@ujs.edu.cn (Z.L.); a.halah@outlook.com (H.A.); amekrema28@hotmail.com (H.M.A.A.)

**Keywords:** bioactive compounds, cold plasma, liquid foods, membrane processing, non-thermal processing, shelf life extension

## Abstract

Consumer demands for fresh and minimally processed liquid foods that support disease prevention and promote health emphasize the need for innovative processing technologies that ensure microbiological safety and preserve bioactive compounds. In addition, consumers are becoming more concerned about the presence of chemical additives in liquid foods. Non-thermal processing technologies, including high-pressure processing, high-pressure homogenization, pulsed electric field, pulsed magnetic field, high-pressure carbon dioxide, ultrasound treatment, radiation processing, ozone processing, cold plasma, and membrane processing, offer excellent prospects for the application in liquid foods. The given technologies aim to retain bioactive properties, deactivate enzymatic activity, and destroy microorganisms, thereby extending the shelf life of liquid foods. Thus, this current review, without a doubt, could be valuable to the liquid food industries and the scientific world by offering great insight into the latest developments in the use of innovative non-thermal processing technologies, which can be employed for shelf life extension and the retention of bioactive compounds in liquid foods. This paper also discusses the challenges faced by the liquid food industry that need to be addressed in future studies.

## 1. Introduction

The current consumer market increasingly demands natural, tasty, fresh, safe, healthier, and organic liquid foods manufactured in an environmentally friendly manner with small carbon footprints and sustainable methods [1]. At the same time, public concerns are increasing about the presence of chemical additives in liquid foods [2]. Liquid foods, such as fruit juices, vegetable juices, milk, coconut water, alcoholic beverages, and non-alcoholic beverages, are rich sources of bioactive compounds, including organic acids, polysaccharides, polyphenols, carotenoids, flavonoids, vitamins, anthocyanins, and dietary fiber [3,4,5,6]. These bioactive compounds exhibit a wide range of potential biological activities, including antioxidant, hepatoprotective, anticancer, anti-aging, cardioprotective, anti-atherosclerotic, antiapoptotic, antiadipogenic, antiangiogenic, cholesterol-modulating, cell-proliferative, and endothelial function-enhancing effects [7]. Liquid foods are widely accepted and well-suited for consumers of all age groups due to their refreshing and hydrating nature, along with their unparalleled appealing sensory attributes, such as color, texture, aroma, and flavor. However, liquid foods contain a high amount of water content and are susceptible to microbial growth and enzymatic spoilage [8]. FDA guidelines state that innovative liquid food processing technologies should achieve a 5 log reduction in the microbial load of pathogenic microorganisms [9]. Thus, liquid foods rich in bioactive compounds, with extended shelf life, and free from chemical additives are needed to maintain the global health status of consumers.

The preservation of liquid foods is a continuous fight to combat pathogenic microorganisms and spoilage enzymes that degrade product quality and compromise the safety for human consumption [10,11]. Conventional thermal processing techniques are widely employed in the industry to target spoilage entities and ensure the preservation of liquid foods. Despite effectively reducing enzymatic activity and microbial load, conventional thermal processing has an adverse effect on the nutritional, sensorial, physicochemical, and delicate bioactive properties of liquid foods [12]. Moreover, modern consumers are increasingly demanding minimally processed liquid foods that offer natural taste, bioactive properties, and considerable shelf stability. Therefore, to address the current preferences, innovative non-thermal technologies have emerged for modern processing of liquid foods [13]. Non-thermal processing technologies offer great prospects for preserving the bioactive properties, sensorial characteristics, reducing enzymatic activity and microbial load, and thereby extending the shelf life of liquid foods [13,14,15,16]. These innovative non-thermal processing technologies offer a plethora of advantages, including better mass transfer rates, lower residence times, improved shelf life, enhanced product functionality, reduced energy consumption, lesser environmental impact, and reduced processing waste, among others [16,17]. Moreover, a combination of innovative non-thermal processing technologies can enhance effectiveness, extend shelf life, and better retain bioactive compounds [15,18]. The novel non-thermal processing technologies are being developed and assessed for their environmental footprints, many of which offer reduced emissions, increased reliability, water savings, energy saving, improved productivity, and higher product quality, thereby minimizing overall environmental impact [19].

Thus, the current review aims to elaborate on the use of selected innovative non-thermal processing technologies for the retention of bioactive compounds and preservation of liquid foods. This document also focuses on technical difficulties associated with the application of non-thermal processing technologies, as well as future perspectives.

## 2. Literature Search and Selection of Studies for Review Article Preparation

The current review discusses and summarizes research in the last six years regarding the application of innovative non-thermal processing technologies for shelf life extension and retention of bioactive compounds in liquid foods. The literature survey was performed using Google Scholar and Scopus by looking up Non-thermal technologies OR High-pressure processing OR High-pressure homogenization OR Pulsed electric field OR Pulsed magnetic field OR High-pressure carbon dioxide OR Ultrasound treatment OR Radiation processing OR Ozone processing OR Cold plasma OR Membrane processing OR Combination of techniques AND Shelf life extension OR Microbial stability OR Liquid food preservation AND Bioactive compounds OR Phytochemicals OR Nutraceuticals AND Liquid foods OR Juices OR Beverages OR Milk for a comprehensive citation search. The inclusion criteria were “Research article”, “English language”, and “Published after 1 January 2020”. The research data from relevant literature were collected and critically discussed. Though most of the cited literature is from the last six years, in some cases, a few older references were included to explain the facts and results that have not been revisited in subsequent studies.

## 3. Basics of Innovative Non-Thermal Processing Technologies

### 3.1. High-Pressure Processing

High-pressure processing plays an imperative role in advanced food processing technologies, offering a significant advantage in quality retention and microbial safety. The food is placed inside a hermetically sealed chamber during high-pressure processing and then exposed to hydrostatic pressure ranged from 100 to 900 MPa to the food sample. The high-pressure processing is governed by three fundamental principles, including the principle of microscopic ordering, the isostatic principle, and Le Chatelier’s principle [20]. The fundamental principle of microscope ordering explains that the increase in degrees of the molecular ordering of a given substance occurs due to an increase in pressure at a constant temperature, which in turn exerts chemical reactions and antagonistic forces on molecular structure. Evidently, the isostatic principle explains that applied pressure is distributed evenly from all directions during compression, irrespective of size, shape, or composition of the product. Likewise, Le Chatelier’s principle reveals that any process in equilibrium, such as conformational change, phase transition, or chemical reaction, which involves a decrease in volume, could be enhanced by application of pressure [21]. During high-pressure processing, covalent bonds in the liquid foods are unaffected, while hydrogen bonds are disrupted since high-pressure processing is a non-thermal technology. The high-pressure processing employs intense pressure on the microbial cell walls, causing irreversible damage, which leads to a reduction in microbial load. The extreme pressure applied during processing also causes enzyme denaturation, thereby preventing enzymatic spoilage [22]. Thus, acceptable pressure limit may be optimized with mild to moderate heat to effectively extend the shelf life along with retention of bioactive compounds in liquid foods.

### 3.2. High-Pressure Homogenization

**Figure 1:**
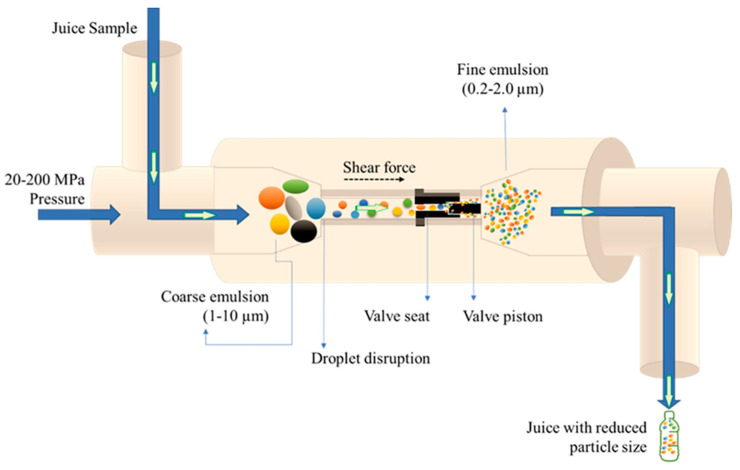
Schematic diagram of high-pressure homogenization of juice. Adapted with permission [28]. Copyright 2022 Elsevier Ltd.

High-pressure homogenization is an innovative technology with diverse applications such as emulsion formation, change in protein structure, disruption of starch granules, improvement of rheological characteristics, enhancement of bioactive properties, inhibition of enzymatic activity, and microbial inactivation in food matrices [23,24,25,26]. The schematic diagram of high-pressure homogenization of juice is depicted in Figure 1. High-pressure homogenization principle depends on cavitation, strong shear forces, and abrupt pressure gradients. The contribution of each aspect to homogenization depends on temperature, pressure, valve geometry, fluid viscosity, equipment size, and valve design. However, turbulent interactions have been collectively recognized as the principal cause of particle fragmentation and subsequent dispersion. The operating pressure of high-pressure homogenization ranged from 20 to 500 MPa. Foods that are exposed to relatively high pressures (200–300 MPa), are classified as ultra-high-pressure homogenization [26,27].

### 3.3. Pulsed Electric Field

The pulsed electric field is one of the promising non-thermal technologies significantly employed for pasteurization of liquid foods. Pulsed electric field is a combination of high-voltage pulses (20 to 80 kV cm^−1^) and electric field that greatly reduces the microbial load present in liquid foods. The strength of the electric field and treatment time are the two key factors that significantly affect the processing efficacy of pulsed electric field. The exposure of pulsed electric field disrupts the cell membrane by inducing electrochemical instability, resulting in release of cell constituents and subsequent microbial death [29]. The pulsed electric field operates on the principle in which a liquid food is pumped through a pulse generating system, where high-voltage input pulses create an electrical potential difference across the liquid food held between two electrodes [30].

### 3.4. Pulse Magnetic Field

The pulse magnetic field is one of the key non-thermal technologies that employs a short burst of high-intensity magnetic field to inactivate enzymes and reduce the microbial load without significantly affecting the sensorial characteristics and nutritional properties of liquid food [31,32]. The pulse magnetic field has an easy-to-operate design, low cost, and a simple frame, which primarily consists of a spiral coil, an operating system, and a pulse magnetic field generator. Tesla meter is employed to measure the intensity of the magnetic field, and the temperature of the sterilization chamber is controlled using a cooling tube. The generator produces a high-intensity pulse magnetic field to kill the microorganisms in liquid food positioned inside the solenoid chamber [33]. The pulse magnetic field processing is performed at ambient pressure and temperature, preserving the heat-sensitive bioactive properties of liquid foods.

### 3.5. High-Pressure Carbon Dioxide

The high-pressure carbon dioxide involves the use of CO_2_ under high pressure to inactivate enzymes and microorganisms present in various food matrices. High-pressure carbon dioxide works by diffusing CO_2_ into the cells of microbe, leading to protein denaturation and disruption of the cell membrane, which collectively contributes to microbial inactivation [34]. The two distinct modes of operation of high-pressure carbon dioxide include dense phase carbon dioxide and supercritical carbon dioxide. In dense phase carbon dioxide, CO_2_ is used in dense or subcritical phase below the temperature of 31 °C, maintaining liquid-like characteristics under high pressure, and effectively inactivating enzymes and microorganisms in liquid foods. Likewise, supercritical carbon dioxide employs CO_2_ at supercritical conditions (73.86 MPa, above 31.06 °C), where it exhibits properties of both a liquid and a gas, efficiently inactivating microbes and enzymes in liquid foods [34,35]. As an alternative to traditional pasteurization methods, high-pressure carbon dioxide processing technology offers the advantage of negligible effect on nutritional and sensory properties of heat-sensitive liquid foods.

### 3.6. Ultrasound Treatment

**Figure 2:**
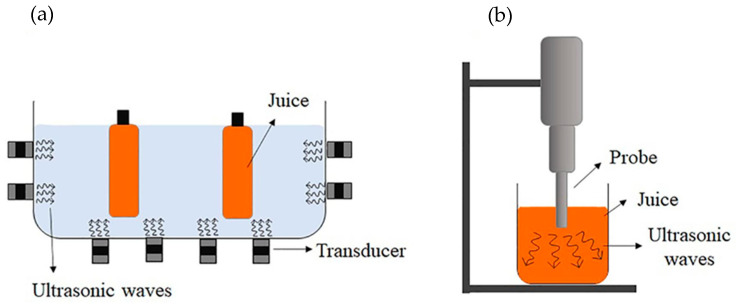
Schematic design of ultrasound application: (**a**) immersion in ultrasonic bath and (**b**) direct application using ultrasonic probe. Adapted with permission [39]. Copyright 2023 Elsevier Ltd.

Ultrasound or sonication can be employed as an alternative to traditional thermal processing for sterilization and pasteurization of liquid foods. Sound waves with frequencies exceeding 20 kHz are used for the immediate transfer of acoustic energy to the product during ultrasound treatment [36]. Ultrasound technology can be employed by either direct application using an ultrasonic probe or by immersing liquid food in an ultrasonic bath. The microbial killing mechanism is primarily due to production of free radicals, thinning of cell membranes, and localized heating. The transmission of ultrasound in a liquid food generates bubble cavitation as a result of pressure fluctuation. The collapse of resultant micro-bubbles induces a localized rise of pressure and temperature. Consequently, the intense high pressure and local energy yield a pasteurization effect without significantly increasing the overall temperature [37,38]. The ultrasound application using ultrasonic bath and ultrasonic probe is depicted in Figure 2a and Figure 2b, respectively.

### 3.7. Radiation Processing

In radiation processing, liquid foods are treated by means of radiation at various wavelengths, including ultraviolet (UV) light, gamma (γ), and electron beam irradiation. The radiation process may be either continuous or discontinuous (pulsed light). UV light processing is highly promising owing to lower cost, lethality to most microbes, ease of use, and dry and cold process compared to other preservation methods. In food processing, UV light spans a wavelength range of 100 to 400 nm, classified into UV-A (320–400 nm), UV-B (280–320 nm), and UV-C (200–280 nm). UV-C radiation is regarded as a germicidal spectrum that is deadly to the majority of microbial strains [40]. Primarily, γ-irradiation inactivates the microorganism through DNA damage. Factors including absence or presence of oxygen, moisture content, and medium composition greatly affect the radiation resistance, specifically in the case of vegetative cells [41]. Evidently, pulsed light employs shot time pulses (100 to 400 ms) of an intense broad spectrum (100 to 1100 nm), with 54% of released energy falling in the range of UV light [42]. Similarly, electron beam employs a low dose of ionizing irradiation to extend the shelf life of the product by eliminating the microbial contamination [43].

### 3.8. Ozone Processing

Ozone is a bluish-colored gas with strong oxidizing properties exhibiting effective enzyme inactivation and strong antimicrobial potential against fungi, bacteria, and their resistance structures. Ozone is generated when free oxygen radicals created by high-energy input reacts with oxygen. Ozone can be generated using various methods, including electrical discharge (corona discharge), electrochemical methods, and the photochemical–ultraviolet radiation method. Ozone easily diffuses through the biological membrane owing to its strong oxidation potential. The ozone gas denatures the protein and microbial DNA by interacting with amino acids, thus resulting in cell death. The ozone reaction with food enzymes or organic compounds can be explained by two mechanisms: (1) oxidation of polyunsaturated fatty acid to form acid peroxides and (2) oxidation of sulfhydryl groups in enzymes, peptides, proteins, and cysteine residues to amino acids. As a result, the reaction of ozone modifies the active sites of enzymes, altering their functions, and resulting in reduced activities. For microbial inactivation, ozone disrupts and oxidizes cellular macromolecules, cell walls, and cytoplasmic membranes, resulting in cell contents leakage and cell lysis. Ozone processing does not pose any harm to the consumer as the residual gas rapidly decomposes into oxygen, and thus it could be employed in the liquid food industry with confidence [44,45].

### 3.9. Cold Plasma

Plasma is a fourth state of matter, consisting of an ionized gas and characterized by actively interacting particles such as atoms, photons, free radicals, negative ions, positive ions, electrons, excited molecules, and nonexcited molecules. In nonthermal plasma, the temperature of the electrons can rise up to 10,000 K, while the entire gas temperature remains close to room temperature, which is why it is referred to as cold plasma. The plasma excitation sources include microwave discharges, helicon discharges, direct current glow discharges (pulsed or continuously), dielectric barrier discharges, corona discharges, capacitively and inductively coupled discharges [46]. Cold plasma generates reactive nitrogen and oxygen species, including nitric oxide radicals, hydroxyl radicals, and singlet oxygen, which influence liquid food quality and microbial inactivation. Cold plasma effectiveness relies upon applied voltage, flow rate, type of feed gas, and plasma source parameters. The mechanism behind microbial inactivation involves the action of reactive species present in cold plasma, which oxidize membrane lipids, disrupt the integrity of cell wall, enhance permeability, cause cellular content leakage, and ultimately lead to cell lysis. Likewise, reactive species present in cold plasma inactivate enzymes by oxidizing amino acid chains, breaking disulfide bonds, limiting metabolic functions, and ultimately causing enzyme denaturation [47]. The cold plasma possesses a strong lethality towards pathogenic microbes and enzyme inactivation, thereby extending the shelf life of the liquid foods.

### 3.10. Membrane Processing

**Figure 3:**
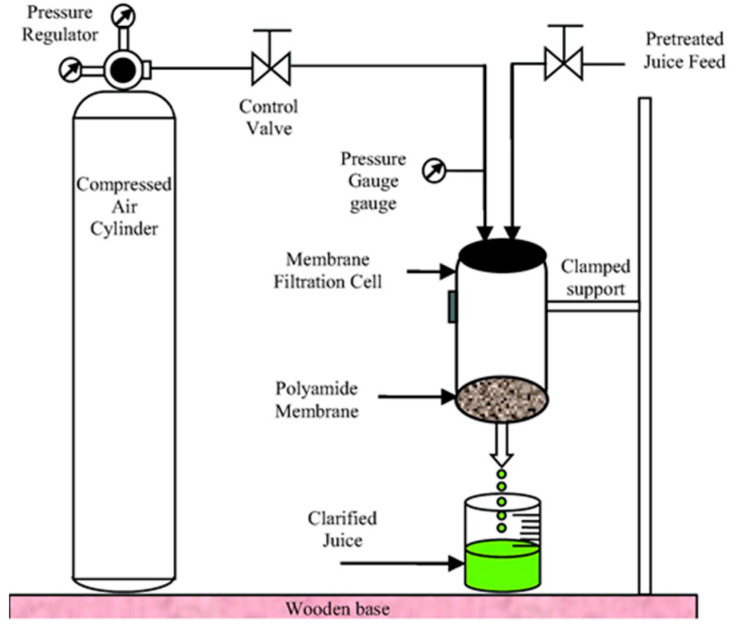
Schematic illustration of membrane processing. Adapted with permission [49]. Copyright 2021 Elsevier Ltd.

Membrane technology is the most widely used method that reduces heat-associated loss of phytochemical and nutritional properties of liquid foods owing to its low temperature operation. The concentration of liquid foods using membrane processing exhibits greater resistance to chemical and microbial deterioration by reducing water activity. The pressure-induced membrane processing, including nanofiltration, reverse osmosis, ultrafiltration, and microfiltration, is extensively employed for the concentration of photoactive compounds in various liquid foods. Ultrafiltration and microfiltration are the predominant processes employed in the bioprocessing industry, and they can separate particles in the approximate size ranges of 1 to 100 µm and 1 to 10 µm, respectively. Ultrafiltration is primarily employed for concentration and clarification purposes, whereas microfiltration is used for clarification of liquid foods. The recent developments in the application of membrane processing include temperature-driven pervaporation, osmotic distillation, and membrane distillation, to name a few, for liquid food processing. The separation primarily occurred through size exclusion based on pore size of the membrane material and solute particles in these processes [48]. The schematic illustration of membrane processing is depicted in Figure 3.

### 3.11. Fundamentals of Using Combined Technologies

The integration of innovative non-thermal processing technologies is fundamentally based on barrier or hurdle theory, which involves the use of multiple preservation factors that work synergistically to inactivate enzymes, prevent microbial proliferation, and slow chemical deterioration. Two or more moderate treatments are integrated in non-thermal processing, rather than a single high-intensity procedure, to target different metabolic pathways or cellular structures while preserving nutritional or sensory quality. Hurdle technologies integrate various approaches with non-thermal methods, including modified atmospheric packaging, chemical preservatives (e.g., sodium benzoate and potassium sorbate), bacteriocins, active packaging, enzymes, antioxidants, thermal or mild heat treatment, and cryogenic cooling, to name a few. The hurdles can be customized according to the type of food, water activity, pH, and intended shelf life. The effectiveness of hurdle technologies primarily relies on compatibility of various components involved and their ease of application in the food industry [16,50].

## 4. Application of Innovative Non-Thermal Processing Technologies

### 4.1. High-Pressure Processing

High-pressure processing is an advanced non-thermal preservation technology that inactivates spoilage enzymes and pathogens in liquid foods. The application of high-pressure processing for shelf life extension and retention of bioactive compounds in liquid foods is reported in Table 1. A study was designed to investigate the effect of high-pressure processing on the quality of sea buckthorn juice. The acquired results revealed that yeast, mold, and total plate count of the juice were ≤10 CFU/mL for 0 to 9 days. Total phenols, total carotenoids, and vitamin C were higher in high-pressure processing-treated samples [51]. High-pressure processed fresh cow milk was investigated for microbial safety and bioactive properties for 60 days of storage at 6 °C. The attained results indicated that high-pressure processed samples successfully retained most of the vitamins and minerals, and shelf life was extended beyond 60 days of storage. Some degradation of vitamins (C, B6, B5, B3, and A), and minerals (zinc and potassium) was documented during 60 days of storage [52]. In another study, high-pressure processed cloudy hawthorn berry juice exhibited a shelf life of at least 150 days. The enzyme activity was ineffectively inhibited compared to thermal processing [53]. Likewise, microbial shelf life of Aronia berry juice was improved by at least five-fold in refrigerated storage and ten-fold at room temperature. The results also documented the successful retention of hydroxycinnamic acid, anthocyanin, and flavonols content for 24 weeks [54]. In another study, high-hydrostatic-pressure processing of fermented pomegranate beverage effectively inhibited microbial growth (<10 CFU/mL) for 42 days of storage. The slight increase was documented for total flavonoids, total phenolic compounds, total anthocyanins, and antioxidant activity compared to pasteurized beverage [55]. Another study reported that high-pressure processed carrot–orange juice blends with a 6 log reduction in *L. innocua* were successfully stored for 28 days. The results reported no significant change in total phenolic, total carotenoid, and ascorbic acid contents [56]. Similarly, raw coconut water processed using high-pressure processing showed no detection of inoculated pathogens, and microbial count remained about 2 log with no detectable sign of deterioration through 120 days of storage [57]. In another study, in vitro bioaccessibility of polyphenols was investigated in high-pressure processed sour cherry juice. The acquired results reported higher total flavonoid bioaccessibility and improved bioaccessibility for most phenolic fractions at 500 MPa. Moreover, high-pressure processed juice indicated lower bioaccessibility for total antioxidant capacity, except at 500 MPa for 20 min conditions compared to control [58]. Another study investigated the impact of high-hydrostatic pressure on enzymes, microorganisms, and anthocyanin content of mulberry juice. The mold, yeast, and total viable count of mulberry juice was <10 CFU/mL. Moreover, high-hydrostatic pressure treatment at 200 MPa/10 min significantly inactivates POD and PPO enzymes and retains the content of anthocyanin [59]. Another study documented that high-pressure processing (>100 MPa) increased the level of ascorbic acid, total phenolics, total flavonoids, gallic acid, catechin, and ferulic acid in *Rosa roxburghii* Tratt juice. Conclusively, the latest work on high-pressure processing highlights its significant potential for extending shelf life and retaining bioactive compounds in liquid foods.

### 4.2. High-Pressure Homogenization

Evident from Table 1, application of high-pressure homogenization has been reported for shelf life extension and retention of bioactive compounds in liquid foods. A study was designed to investigate the effect of ultra-high-pressure homogenization on microorganisms and bioactive compounds of pear juice. The attained results revealed that ultra-high-pressure homogenization treatment successfully reduced bacteria, yeast, and mold count. Moreover, total phenolic content and antioxidant activity were increased after the treatment [61]. In another study, high-pressure homogenization reduced the total plate count and yeast and mold by 4 log_10_ and 3 log_10_ in mixed juice (carrot, apple, peach), respectively [62]. Evidently, ultra-high-pressure homogenization treatment at 250 MPa achieved more than 5 log CFU/mL reduction for *B. subtilis* and *B. pumilus* spores in sheep and cow milk [64]. In another study, ultra-high-pressure homogenization treatment at 200 MPa for three cycles greatly increased the shelf life of kiwifruit juice for more than 40 days at refrigeration storage and increased total phenolic content availability [63]. A study explored the ultra-high-pressure processing treatment impact on immunoglobulin preservation and microbial safety in human milk. The acquired results revealed that treatment at 200 MPa achieved a >5 log lethality for *Escherichia coli*, *Franconibacter helveticus*, and *Listeria innocua*. In addition, no significant reduction was observed in basal concentration of immunoglobulin (sIgA, IgG, or IgM) [65]. In another study, continuous-flow high-pressure homogenization treatment at 300 MPa, 4°C, and 1.5 L/min yielded 54% more anthocyanins after 45 days of storage compared to control (HTST). In addition, lower PPO activity was also recorded in blueberry juice [66]. Likewise, high-pressure homogenization treatment at 150 MPa successfully preserved the total phenolic content and antioxidant capacity (DPPH-ARA, FRAP, and ABTS-ARA) in pomegranate juice [67]. Another study reported that combined juice (peach and carrot) treatment at 200 MPa retained more concentration of polyphenols and carotenoids [68]. The findings of the aforementioned studies indicate that high-pressure homogenization can be employed for shelf life extension and preservation of bioactive compounds in liquid foods.

### 4.3. Pulsed Electric Field

The application of pulsed electric field for shelf life extension and retention of bioactive compounds in liquid foods is reported in Table 1. A study was designed to investigate the impact of pulsed electric field on blood orange juice for shelf life extension and retention of bioactive compounds. The findings of the study reveal that pulsed electric field significantly inactivated the microbes and extended the shelf life (15–20 days) of blood orange juice. The bioactive compounds, including total phenolic content, anthocyanins, ascorbic acid, and individual flavanones, were successfully preserved [69]. Another study investigated the combination of antimicrobial caps and pulsed electric field for orange juice shelf life extension. The results yielded the lowest mold and yeast populations, and no significant differences in vitamin C and total phenolic compounds were detected after combined treatments. The stability of orange juice was maintained for 5 weeks at 10 °C, although vitamin C was lost [70]. Another study reported that pulsed electric field-treated Shalgam juice exhibits lower lactic acid bacteria (∼3% counts) and total aerobic mesophilic bacteria (∼9% counts) after 70 days of storage. The insignificant effect of pulsed electric field was recorded for antioxidant activity and total phenolic content during storage [71]. Likewise, pulsed electric field-treated (18 kV/cm, 200 Hz) pomegranate fermented beverage was recorded with reduced microbial load (4 log cycle) during storage. The slight reduction was observed in anthocyanins, phenolic compounds, antioxidant capacity, and flavonoids [72]. Another study reported that pulsed electric field successfully inactivated PME enzyme up to 43.03% in kiwi–carrot juice. The degradation of ascorbic acid and phenolic compounds was also documented at optimal conditions [73]. Likewise, pulsed electric field combined with natural preservatives (natamycin, and tea polyphenols) improved the inactivation of *S. cerevisiae*, prolonged shelf life, and better retained vitamin C concentration in cantaloupe juice [74]. Another study investigated the impact of pulsed electric field on milk-based beverages and reported that beverage treatment at 80 pulses, with no preservative, allowed storage for up to 6 days at 5 °C [75]. The study investigated the impact of pulsed electric field on bioactive compounds of Sohiong fruit juice. The acquired result revealed a significant increase of 11%, 20%, 12%, and 89% for total anthocyanin content, total phenolic content, DPPH inhibition, and ascorbic acid, respectively [76]. Another study reported that a combination of pulsed electric fields with antibacterial agents (nisin, carvacrol, and heptyl paraben) successfully reduced the growth of *Alicyclobacillus* spp. and maintained the organic acids in apple juice [77]. In conclusion, pulsed electric field offers great potential for controlling microbial proliferation, inactivating enzymes, and maintaining the integrity of bioactive compounds in liquid foods.

### 4.4. Pulsed Magnetic Field

Evident from Table 1, application of pulsed magnetic field has been reported for shelf life extension and retention of bioactive compounds in liquid foods. A study was designed to investigate the impact of pulsed magnetic field on enzymatic and microbial inactivation and bioactive compounds of orange juice. The acquired results revealed that pulsed magnetic field reduced the activities of yeast, mesophilic bacteria, and mold by maximum log reduction of 0.36 and 0.61 at 7 T with 30 pulses and 20 pulses, respectively. The partial inactivation was reported for POD and PME activities. The slight reduction was documented for antioxidant capacity (ABTS, and DPPH), phenolic compounds, and ascorbic acid [78]. Another study indicated a superior antibacterial effect of pulsed magnetic field against *E. coli* O157:H7 in tomato, lettuce, carrot, and cucumber juices [79]. Likewise, another study reported that a combination of Litseacubeba essential oil and pulsed magnetic fields successfully maintained the number of *E. coli* O157:H7 close to 1 log CFU/mL in cucumber, carrot, spinach, and bitter gourd juices [82]. In another study, cloudy apple juice was treated with pulsed magnetic field for enzymatic inactivation and preservation of bioactive compounds. The findings of the study revealed that PPO, POD, and PME activities were successfully inhibited, and a significant decrease in ascorbic acid was observed. In addition, no change was recorded in DPPH activity and phenols [80]. Likewise, pulsed magnetic field successfully decreased the bacterial load to 1.43 × 10^4^ CFU/mL in orange juice at 4 T, 15% juice, and 15 min. Also, mold and yeast counts reduced to 1.68 × 10^4^ CFU/mL at 4 T, 20% juice, and 15 min [81]. Collectively, the findings demonstrate that pulsed magnetic fields hold significant potential for extending shelf life and preserving bioactive compounds in liquid foods.

### 4.5. High-Pressure Carbon Dioxide

The application of high-pressure carbon dioxide for shelf life extension and retention of bioactive compounds in liquid foods is documented in Table 2. A study was designed to investigate the impact of supercritical carbon dioxide in sugarcane juice. The results yielded a successful reduction for yeast, molds, mesophiles, and lactic acid bacteria. The reduction in POD and PPO ranged from 0.27 to 41.42% and 3.51 to 64.18%, respectively. The authors stated that supercritical carbon dioxide combined with mild temperature significantly preserves the sugarcane juice [83]. In another study, dense-phase carbon dioxide completely inactivated the pathogens, PPO, and POD activities in mango in syrup. The shelf life of the product was documented to be less than 86 days. In addition, higher content of total phenols and vitamin C was recorded after the treatment [84]. Likewise, supercritical carbon dioxide treatment produced a microbiologically stable pomegranate juice that remained stable for up to 28 days of storage at 4 °C. The total phenol content was successfully maintained, while the antioxidant activity decreased during storage [85]. Another study documented lower enzymatic activity in peach, apple, and pear juices following high-pressure carbon dioxide treatment. However, the results also yielded a slight decline in phenolic compounds [86]. A study explored the supercritical carbon dioxide processing of beverages containing *Pfaffia glomerata* root extract and apple juice. The attained results indicate that supercritical carbon dioxide treatment successfully maintained the fructooligosaccharide content in the prepared beverage [87]. Similarly, another study reported that supercritical carbon dioxide successfully enhanced the stability of total anthocyanins, vitamin C, and antioxidant capacity in blackcurrant juice [88]. In conclusion, high-pressure carbon dioxide demonstrates considerable potential to suppress microbial growth, deactivate enzymes, and maintain bioactive compounds in liquid foods.

### 4.6. Ultrasound Treatment

Ultrasound, as a novel non-thermal technology, has been widely adopted in the agri-food industry [109]. The liquid food treatment with sonication significantly reduced the microbial population and increased the bioactive compounds. However, food matrix heterogeneity could lower the inactivation efficiency of ultrasound treatment [110]. As evident from Table 2, the application of ultrasound treatment has been reported to extend the shelf life and retain bioactive compounds in liquid foods. A study was designed to investigate the effect of high-intensity ultrasound (HIUS) treatment on Jabuticaba juice. The acquired results revealed that HIUS effectively inactivated PPO, POD, and PME activities. In addition, HIUS treatment successfully retained anthocyanin, exhibited phenolic stability, increased gallic acid (up to 55%), and maintained ellagic acid levels. The study also documented up to 40% increase in cyanidin-3-O-glucoside content [89]. In another study, *Opuntia stricta* var. *dillenii* extract-enriched Brazil nut beverage was pasteurized using ultrasound treatment. The attained results highlight that ultrasound treatment (80% amplitude, 12 min) effectively achieved a ≥5 log reduction in *E. coli* and remarkably preserved the total phenolic, total flavonoid, and total betaxanthin contents [90]. Likewise, dual-frequency energy-gathered ultrasound pretreatment (DEUP) and flat-sweep-frequency dispersive ultrasound pretreatment (FSDUP) increased the storage period of strawberry clear juice by up to 14 and 21 days, respectively. Moreover, the reduction in DPPH activity, ascorbic acid, anthocyanins, flavonoids, and phenols was documented during storage [91]. Another work reported that ultrasound (700 W) combined with phloretin (300 μg/mL) resulted in reduction in *Staphylococcus aureus* (6.6 log CFU/mL) and *Escherichia coli* (6.5 log CFU/mL) and maintained the quality of cloudy apple juice after 14 days of storage [92]. Similarly, another study revealed that thermosonication greatly inactivated the microbes and enhanced the content of total phenols (34.06%), flavonoids (33.94%), ascorbic acid (36.64%), and lycopene (42.13%) in freshly squeezed tomato juice. Thermosonication also maintained a higher antioxidant capacity compared to thermal pasteurized sample [93]. In another study, ultrasound treatment considerably decreased the microbial population in black carrot juice and increased the antioxidant properties, ascorbic acid, and phenolic content [94]. Another study reported that thermosonication significantly reduced the microbial load (≤1 log CFU/mL) and inactivated POD and PPO activities to 0.018 and 0.31 Abs min^−1^ in spinach juice, respectively. Additionally, significant improvements were documented for total flavonols, total phenolics, total flavonoids, anthocyanins, chlorophyll, carotenoids, and antioxidant activities (FRAP and DPPH assays) [95]. Likewise, melon juice treated with high-intensity ultrasound yielded a reduced microbial load. The increase of up to 42% was recorded for total carotenoids, while a 33% decrease was observed for total phenolic content. In addition, DPPH, ABTS, and FRAP capacities were enhanced after ultrasound treatment [96]. Another study revealed that ultrasound treatment of Kinnow fruit juice enhanced the total phenolic content, whereas vitamin C and antioxidant activity were decreased [97]. Similarly, a study reported that thermosonication (SDU-60 °C) reduced the microbial load, inactivated the PPO enzyme, and reduced the loss of total phenolic content (approximately 70%) [98]. In another study, thermosonication (60%, 86 °C) delivered excellent retention of antioxidant activity, phenolic compounds, and anthocyanins [99]. The studies reviewed indicate that ultrasound treatment can be employed to prolong the shelf life and preserve the bioactive compounds in liquid foods. Moreover, ultrasound treatment can markedly influence the sensory attributes, including texture, color, aroma, and flavor of liquid foods owing to its chemical, cavitation, and mechanical effects. A previous study indicated that temperature-controlled ultrasound treatment improved the odor, cloudiness, and appearance of cloudy apple juice [111]. Another study revealed that thermosonication improved the color, flavor, and aroma of tomato juice [93]. Likewise, a study documented that thermosonication had a positive effect on color and flavor of strawberry juice [98]. Nevertheless, another study reported that high-intensity ultrasound treatment for an extended period of time slightly decreased the color quality of soybean milk, possibly due to a rise in brown pigments and accelerated Millard reaction. In addition, ultrasound treatment decreased the off-flavors of soybean milk for the first seven minutes of treatment before increasing after nine minutes, presumably due to a variation in volatile compounds [112]. Therefore, critical optimization of ultrasound parameters is required to maintain desire sensory attributes, thus assuring commercial adoption and consumer acceptance.

### 4.7. Radiation Processing

The application of radiation processing for shelf life extension and retention of bioactive compounds in liquid foods is documented in Table 2. A study was designed to investigate the impact of ultraviolet (UV) processing on shelf life and bioactive compounds of coconut water. The acquired results revealed that the UV-treated sample achieved 12 days of shelf life. A 5 log_10_ reduction in *E. coli* and 60% inactivation of POD activities was achieved. In addition, ascorbic acid and phenolics were decreased with an increase in fluence [100]. Another study reported that UV-C treatment extended the shelf life of cold brew coffee up to 14 days (4 °C). Moreover, UV-C-treated samples presented 1.4 and 1.6 times higher contents of chlorogenic acid and total polyphenols, respectively, compared to the control on the 14^th^ day of storage [101]. Likewise, UV-C radiation combined with nisin produced microbiologically stable coconut water up to 24 days of refrigerated storage. The microbial population remained below 10^6^ CFU/mL up to 24 days of storage. A consistent reduction in total flavonoids (20%), total phenolic compounds (18%), and antioxidant activity (50%) was documented [102]. Pulsed light is a non-thermal innovative technology that offers great prospects to reduce microbial contamination [113,114] and degrade aflatoxin B_1_ and B_2_ [115]. A study on lactic acid fermented mulberry juice treated with pulsed light documented a decrease in microbial load (1.02 ± 0.04 log_10_ CFU/mL) and slightly reduced anthocyanin content at exposure time of 8 s [103]. Another study reported that pulsed light at optimal conditions increased the total flavonoids (489.12 mg/100 mL) and total phenolics (982.30 mg/100 mL), whereas no difference in total anthocyanin content was recorded compared to control [104]. Likewise, a study revealed that pulsed light successfully reduced *E. coli* and slightly affected total phenolic, ascorbic acid, and anthocyanin content in freshly squeezed grape juice [105]. The study was designed to investigate the impact of antimicrobial formulation and γ-irradiation on microbial inactivation and bioactive compounds of mother’s milk. The acquired results revealed that γ-irradiation combined with the third and fourth antimicrobial formulations eliminated all tested pathogens and significantly retained immunoglobulins [106]. Another study revealed that sulforaphane content in broccoli sprout juice was reduced with an increase in electron beam irradiation dose [107]. Likewise, another study reported that electron beam irradiation (2 kGy) significantly reduced the total microbial count by 6 log in raw goat milk [108]. Thus, radiation processing exhibited strong potential for controlling microbial growth, deactivating enzymes, and maintaining the integrity of bioactive compounds in liquid foods. Additionally, the impact of radiation processing techniques, including UV light, γ-irradiation, pulsed light, and electron beam irradiation on the sensory attributes of liquid foods, remains a critical concern for commercial adoption and consumer acceptance. However, previous studies indicate that radiation processing significantly improved the sensory properties in liquid foods [101,104,107,116]. Maintaining a balance between sensory attributes, microbial safety, and retaining bioactive compounds is crucial for the broader application of radiation processing in the liquid food industry.

### 4.8. Ozone Processing

As evident from Table 3, application of ozone processing has been reported for shelf life extension and retention of bioactive compounds in liquid foods. A study was designed to investigate the effect of ozone processing on the shelf life of pasteurized skim milk. The attained results reveal that total viable count of pasteurized skim milk was greatly reduced and shelf life was extended up to 15 days of storage [117]. Likewise, another study documented that ozonation produced a microbiologically stable juice that remained stable during 3 months of storage. In addition, an increase in total flavonoids, phenolics, and antioxidant potential was also recorded after ozone processing [118]. In another study, microbial population of ozone-processed Kinnow juice was reduced along with moderately enhanced total flavonoids, phenols, antioxidant activity, and DPPH activity [119]. Likewise, ozone treatment reduced the microorganism in unclarified (3.150 log) and clarified (3.466 log) watermelon juice. In addition, PME activity was not altered, and lycopene, ascorbic acid, and total phenolic content were degraded during processing [120]. In another study, microbial load was reduced in mango nectar along with an increase in vitamin C, total carotenoids, and total phenolic value after ozone processing [121]. Similarly, ozone treatment reduced the PPO activity with longer exposure time and reduced the total phenol and flavonoid content in sugarcane juice [122]. These findings reveal that ozone processing can be utilized for shelf life extension and preservation of bioactive compounds in liquid foods. Ozone, being a potent oxidizing agent, may pose concerns regarding sensory characteristics of liquid foods. A previous study documented that ozone-treated nectar had the best appearance, odor, and taste characteristics along with microbial quality [121]. Another study reported that ozone treatment markedly retained the appearance, taste, smell, and mouthfeel in skim milk stored at 4 °C [117]. A study also documented that sensory qualities, including color, appearance, texture, flavor, and overall acceptability of ozone-treated Kinnow juice was inferior compared to the control samples [119]. Consequently, the use of ozone treatment in liquid foods should be optimized to maintain desire sensory attributes, thereby assuring consumer acceptance and market adoption.

### 4.9. Cold Plasma

The application of cold plasma for shelf life extension and retention of bioactive compounds in liquid foods are documented in Table 3. A study was designed to investigate the effect of cold plasma on storage stability and shelf life of kiwifruit juice. The attained results revealed that the shelf life of optimized cold plasma-treated samples was 60, 80, and 100 days at 25 °C, 15 °C, and 5 °C, respectively. However, more than 50% ascorbic acid loss was also reported [123]. Likewise, another study reported that the shelf life of optimized cold plasma-treated pineapple juice was 25, 50, and 90 days at 25 °C, 15 °C, and 5 °C, respectively. The cold plasma-treated sample was recorded with microbial population of <1 log CFU/mL, though bioactive substances were reduced during storage [124]. Similarly, optimized non-thermal plasma treatment of pineapple juice extended the shelf life and retained ascorbic acid, total phenol, antioxidant capacity, and flavonoids. The microbial population remained <1 log_10_ CFU/mL, and over 90% enzyme inactivation was documented [125]. Another study revealed that atmospheric cold plasma produced microbiologically stable red dragon fruit juice for 28 days at 4 °C and increased the total phenols (37.59%). In addition, antioxidant levels and flavonoids were reduced at longer storage periods and exposure times [126]. Evidently, a study revealed a successful 5 log reduction in *E. coli* in NFC apple juice for 30 days of storage. Moreover, continuous cold plasma greatly preserved ascorbic acid, phenolics, and antioxidant properties as well as chlorogenic acid, l-epicatechin, and cianidanol [127]. The findings of another study reveal that cold plasma reduced the microbial load, enhanced the microbial stability, and prolonged the shelf life of raw buffalo milk [128]. Likewise, atmospheric cold plasma reduced *A. acidoterrestris* spores; however, mold and yeast counts increased in cloudy apple juice during storage. The antioxidant activity remained stable, whereas total phenolic content increased post-storage [129]. In another study, atmospheric cold plasma successfully reduced the colony count in coconut milk [130]. Another study reported that cold plasma successfully inactivated enzymes and microbes in orange juice. However, it also led to degradation of ascorbic acid content [131]. Likewise, application of atmospheric cold plasma considerably degraded PPO, POD, and anthocyanins in camu-camu juice. However, concentration of ascorbic acid was increased at higher excitation frequencies [132]. Conclusively, cold plasma shows considerable promise for controlling microbial proliferation, inactivating enzymes, and retaining bioactive compounds in liquid foods.

### 4.10. Membrane Processing

As evident from Table 3, application of membrane processing has been reported for shelf life extension and retention of bioactive compounds in liquid foods. A study explored the optimization of microfiltration for shelf life extension to maximize the antioxidant compounds. The attained results reveal that microfiltration successfully reduced the microbial load in watermelon juice, providing a shelf life comparable to pasteurized juice (14 days). Microfiltration also effectively concentrated flavonoids, lycopene, and phenolic compounds [133]. Another study reported that ultrafiltered cashew apple juice was markedly stored for 12 weeks. At the optimum condition, tannic acid was increased, whereas ascorbic acid and total polyphenol content were reduced during storage [134]. A study explored the potential of ultrafiltration of sugarcane juice for shelf life extension and retention of bioactive compounds. The acquired results revealed a 6 log and 3-fold reduction in bacterial count and oxidative enzyme, respectively. The prepared clarified juice was stable for 9 weeks (4 °C) with 80% recovery of polyphenols [135]. Evidently, another study reported that microfiltration reduced the microbial population and increased the shelf life of Shalgam juice when stored at 4 °C [136]. Likewise, microfiltration combined with enzymatic treatment yielded microbiologically safe prickly pear juices for 90 days of storage (4 °C) [137]. Another study reported that microfiltration reduced the fermentation microorganism (53.93%) in Kombucha drinks [138]. Similarly, the results of another study revealed that microfiltration combined with centrifugation preserved the 87% ascorbic acid content in cashew apple juice [139]. In conclusion, the findings of the above-mentioned studies show that membrane processing can be employed to extend the shelf life and retain bioactive compounds in liquid foods.

### 4.11. Other Non-Thermal Processing Technologies

High-voltage electrospray, induced electric field, moderate electric field, elongated electrode moderate electrical field, and high-voltage electric field were successfully employed for microbial load reduction, inactivation of enzymes, and preservation of bioactive compounds in liquid foods. A study reported that high-voltage electrospray technology successfully achieved bacterial inactivation (2.31 log) and extended the shelf life of raw bovine milk to 14 days at 4 °C at 80 kV of voltage compared to control [140]. Likewise, in another study, high-voltage electrospray reduced the total number of colonies to 2.03 log CFU/mL in honey raspberry wine and showed decent stability during 84 days of storage at 4 °C [141]. Likewise, high-voltage electrospray-treated cabbage juice documented POD and PPO relative activities of 6.68% and 5.24%, respectively [142]. In another study, high-voltage electrospray treatment successfully reduced the total number of bacteria to 2.03 log CFU/mL in honey raspberry wine and retained the contents of anthocyanins and ascorbic acid at 77.64% and 74.36%, respectively. In addition, SOD activity remained unchanged after processing [143]. In another study, induced electric field successfully ensured microbial safety and preserved the total flavonoids, phenolics, and anthocyanins in bayberry juice [144]. Likewise, moderate electric field successfully achieved 3.43 log reduction of microorganisms at 175 kV for 15 min and extended the shelf life of grape juice [145]. Similarly, elongated electrodes moderated electric field at optimal conditions (0.018 kg/s MFR, 30 V/cm EFI), decreased the microbial count by 2.07 log cycles, and yielded 25% longer shelf life than thermal pasteurization [146]. Another study reported that high-voltage electrostatic field reduced PPO and PME activity by 67% and 43.7% in apple juice during 7 days of cold storage, respectively [147]. The findings of the aforementioned studies indicate that these innovative non-thermal technologies can be employed to inactivate enzymes, reduce the microbial load, and preserve bioactive compounds in liquid foods.

### 4.12. Combination of Various Techniques

The limitations associated with individual treatment can be addressed using a combination of innovative non-thermal processing technologies to enhance the shelf life and retention of bioactive compounds. The application of combined technologies for shelf life extension and retention of bioactive compounds in liquid foods is documented in Table 4. A study was designed to investigate the effect of ozonation and ultrasonication on the preservation of strawberry–cantaloupe functional drink incorporated with orange peel and *Spirulina platensis* extracts. The acquired results reveal that a combination of ozone and ultrasonication treatment extends the shelf life for two months at 4 °C and retains the bioactive compounds, including total phenolics, total flavonoids, total carotenes, ascorbic acid, DPPH, ABTS, and FRAP assays [148]. Another study revealed that a combination of pulsed electric field (2.19 min) and high-power ultrasound treatment (7.48 min) markedly improved anthocyanin stability in strawberry juice during 7 days of storage at 4 °C [149]. In another study, a combination of ozone processing and ultrafiltration achieved 7 log bacteria, 91% peroxidase, 85% polyphenoloxidase, and 5.2 log mold and yeast inactivation in sugarcane juice. Caffeic acid was decreased by 26.7% and vitexin and its derivatives did not reduce significantly. The attained results reveal that combined treatment produced a microbiologically stable juice, which can be stored up to 90 days [150]. Evidently, a combination of microfiltration and UV-C reduced the bacterial load by approximately 5 log and extended the refrigerated shelf life of skim milk for 40 days. Bioactive serum proteins such as lactoperoxidase, immunoglobulins, and lactoferrin were not damaged during processing [151]. Likewise, a combination of high-powered ultrasound and pulsed electric field documented that hydroxycinnamic acids and total phenols were the most stable bioactive compounds in strawberry juice. The results also revealed that condensed tannin and flavonol stability depend on duration of both treatments [152]. Similarly, integration of ultrafiltration and high-pressure processing retained the total flavonoids (84.19%) in chestnut rose juice; however, significant reductions in kaempferol, quercetin, catechin, and myricetin were documented [153]. Another study employed the integration of ultrasonication and pulsed electric field on spinach juice and achieved PPO and POD inactivation to 0.011 Abs/min and 0.025 Abs/min, respectively. The combined treatment attained the highest value of vitamin C, carotenoids, flavonols, anthocyanins, flavonoids, antioxidant capacity, DPPH, phenolic, and total chlorophyll [154]. Likewise, another study achieved a 3 log reduction in total plate count, 80% POD, and 92% PPO activity in sugarcane juice using a combination of membrane processing and pulsed electric field. The combined treatment also retained ascorbic acid content [155]. Another study documented that a combination of high-pressure processing and pulsed electric field reduced the microbial load, enhanced the shelf life, and increased the antioxidant activity and antioxidant contents in Concord grape juice [156]. Evidently, a combination of microfiltration and ultrasonication successfully removes the bacteria, extends the shelf life for at least 40 days, and retains the bioactive proteins including IgG, IgA, and IgM [157]. Another study reported that a combination of microfiltration and high-pressure processing reduced the total bacterial and *E. coli* count to 4 log units and successfully preserved whole milk [158]. Likewise, ultrafiltration combined with ozonation reduced the spoilage rate and phenolic content degradation in sugarcane juice [159]. Conclusively, a combination of innovative non-thermal technologies offers significant potential for controlling microbial proliferation, deactivating enzymes, and maintaining the integrity of bioactive compounds in liquid foods.

## 5. Technical Challenges and Future Prospects

The innovative non-thermal processing technologies extend the shelf life of liquid foods without the need for additives or preservatives while effectively retaining the bioactive properties. The application of non-thermal technologies as an assisting/combined technology facilitates the development of faster and feasible processes compared to conventional methods, and it can deliver various advantages, including lower cost, reduced processing time, improved mass transfer, higher product quality, and increased extraction yield. However, the implementation of innovative non-thermal processing technologies is hindered by various technical challenges. High-pressure processing has been adopted at a commercial scale by the liquid food industry. Despite this, the main limitations of this technology are high capital expenditure and limited throughput. For instance, processing cost for pasteurizing orange juice is seven times higher than that of the thermal pasteurization method [160]. In addition, inappropriate pressure conditions may lead to survival of enzymes and microorganisms, and processing should be conducted at low temperature in order to protect the heat-sensitive bioactive properties. Similarly, high-pressure homogenization technology is accompanied by challenges such as higher equipment cost, more energy consumption, heat generation during processing, flavor or color change, and limited effectiveness on viscous liquids. Likewise, pulsed electric field has limitations of higher capital cost, lower penetration depth, formation of bubbles in the products, non-uniform distribution of electric field, and challenges in continuous flow system design. The treatment chamber of pulsed electric field also possesses challenges for unclogging and cleaning the system. The ionic strength and pH of the liquid food might impact the conductivity of the medium and alter the distribution of electric field and hence the efficacy of pulsed electric field. Likewise, high solid content and viscosity impede the energy transfer, reduce the uniformity of electric field, and impair microbial inactivation. Evidently, pulse magnetic field proved to be an eco-friendly and time-saving technology; however, test repeatability is not ideal owing to raw materials, growth state of microorganism, and type of equipment, to name a few. In addition, pulsed magnetic field possesses significant challenges in scaling up for industrial processing. Similarly, the efficacy of high-pressure carbon dioxide can be enhanced by achieving uniform CO_2_ homogenization. The high viscosity and °Brix of liquid foods still influence the treatment outcomes owing to limited CO_2_ diffusion. The formation of bicarbonates and carbonic acid as chemical residues during processing should be further explored. Advanced equipment with improved CO_2_ homogenization using enhanced agitation or microbubble systems is crucial to make high-pressure carbon dioxide more scalable and effective for industrial use. Likewise, ultrasound treatment possesses challenges for process optimization of various liquid food matrices, metal contamination from ultrasound horns, and higher cost for equipment. High-power equipment is required for significant inactivation of microorganisms and enzymes. In addition, microbial sensitivity to mechanical stress can be influenced by the pH of the liquid foods. The cavitation bubble formation can be disrupted by high viscosity and solid content, which reduces effectiveness. Moreover, radiation processing exhibits limitations related to regulatory aspects, low penetration power, and limited consumer acceptance. Likewise, ozone processing causes changes in acidity and pH of liquid foods, corrosion at high concentrations and limited penetration in viscous or cloudy liquids. Evidently, cold plasma processing can result in product discoloration, enhanced acidity, oxidation reaction, change in pH value, and lower penetration depth. Likewise, membrane fouling decreases the process efficiency and results in reduced flux and high maintenance. Additionally, variability in the origin of liquid foods can markedly influence their chemical composition, such as protein, fat, carbohydrates, minerals, and organic acids, as well as their physical properties, which includes density, viscosity, and optical characteristics. The differences in composition and physical properties of liquid foods significantly affect the interaction mechanism and overall efficiency of innovative non-thermal processing technologies [161,162]. Future studies should investigate the design modification and environmental impact of non-thermal processing technologies to accomplish economic viability. The suitable optimization of given techniques is required by employing either individual or assisted/combined processes for retention of bioactive properties of liquid foods. Moreover, outgrowth and germination potential of surviving spores after non-thermal treatment should be investigated for better shelf life extension of liquid foods. Future work is required to comprehend the resistance of Gram-negative and Gram-positive bacteria in relation to the thickness of the cell wall and cell membrane. Future research should incorporate energy expenditure assessments, cost–benefit analysis, and life cycle assessment in comparison to traditional technologies to examine the environmental and economic viability of innovative non-thermal processing technologies. Additionally, innovative non-thermal technologies should be integrated with artificial intelligence and machine learning algorithms to enhance their implementation in the food industry. The research should also focus on developing advanced membrane materials to further improve process efficiency and product quality. The milk and juice processing industries stand to benefit most from the implementation of innovative non-thermal processing technologies, facilitating extended shelf life and enhanced retention of bioactive compounds. Nevertheless, extensive utilization of non-thermal processing in the liquid food industries has yet to thrive, and additional research is required to elucidate specific sterilization mechanisms, overcome the technical challenges, and broaden application domains.

## 6. Conclusions

The retention of bioactive compounds and microbial safety are two essential and parallel consumer requirements for safety and acceptability of liquid foods. The innovative non-thermal processing technologies effectively retain the bioactive properties, inactivate PPO, POD, and PME enzymes, and reduce microbial loads, thereby extending the shelf life of liquid foods. The combination of innovative non-thermal technologies demonstrates immense potential in achieving microbial reduction standards while retaining bioactive compounds in liquid foods. However, high cost remains a barrier and prevents the diffusion of these technologies into practice at the industrial level. It is noteworthy to state that thorough investigation of underlying mechanisms and commercially feasible non-thermal processing technologies should be developed for large-scale application based on techno-economic aspects.

## Figures and Tables

**Table 1:** Application of high-pressure processing, high-pressure homogenization, pulsed electric field, and pulsed magnetic field for shelf life extension and retention of bioactive compounds in liquid foods.

Liquid Food	Processing Conditions	Key Findings	Ref.
High-pressure processing			
Sea buckthorn juice	500 MPa for 5 min	Total plate count, mold, and yeast count were ≤10 CFU/mL for 0 to 9 days. Total phenols, total carotenoids, and vitamin C were higher in high-pressure processing.	[51]
Cow milk	600 MPa for 10 min	Successfully retained all vitamins, minerals, and extend the shelf life beyond 60 days of storage.	[52]
Cloudy hawthorn berry juice	300 and 600 MPa for 2 and 6 min	High-pressure processing enhanced the shelf life for at least 150 days.	[53]
Aronia berry juice	600 MPa for 5 min	Improved microbial shelf life by at least 5 times at refrigerated storage and 10 times at room temperature. Successfully retained hydroxycinnamic acid, anthocyanin, and flavonols content for 24 weeks.	[54]
Fermented pomegranate beverage	500, and 550 for 10 min, and 600 MPa for 5 min	High hydrostatic pressure successfully contained microbial growth for 42 days of storage. Total flavonoids, total phenolic compounds, total anthocyanins, and antioxidant activity were slightly increased after processing.	[55]
Carrot-orange juice blends	200, 300, and 400 MPa for 1 to 5 min	High-pressure processing attained higher than 6 log reduction in *L. innocua*, and blends were more stable during 28 days of storage. Total phenolic, total carotenoid, and ascorbic acid content did not change significantly.	[56]
Raw coconut water	593 MPa for 3 min	No detection of inoculated pathogens was reported and microbial count remained about 2log with no detectable sign of deterioration through 120 days of storage.	[57]
Sour cherry juice	300, 400, and 500 MPa for 5, 10, and 20 min	Reported higher total flavonoid bioaccessibility and improved bioaccessibility for most phenolic fractions at 500 MPa. Revealed lower total antioxidant capacity except at 500 MPa for 20 min conditions.	[58]
Mulberry juice	200, 400, and 600 MPa for 10, 20, and 30 min	Treatment at 200 MPa/10 min significantly inactivated PPO and POD enzymes and retained anthocyanin content.	[59]
Rosa roxburghii Tratt juice	100, 200, 300, 400 and 500 MPa for 5,10, 15, 20 and 25 min	High-pressure processing (>100 MPa) increased the level of ascorbic acid, total phenolics, total flavonoids, gallic acid, catechin, and ferulic acid.	[60]
High-pressure homogenization			
Pear juice	50, 100, 150, and 200 MPa at 4, 20, 30, 40, 60, and 80 °C	Ultra-high-pressure homogenization treatment reduced the bacteria, yeast, and mold count, and total phenolic content and antioxidant activity was increased.	[61]
Mixed juice (carrot, apple, peach)	25, 100, 140, 180 MPa, Pass 1 and 2, inlet temperature 25 °C, and 40 °C	High-pressure homogenization reduced total plate count, yeast, and mold by 4 log_10_ and 3 log_10_, respectively.	[62]
kiwifruit juice	200 MPa for 2 and 3 cycles	Treatment at 200 MPa for 3 cycles greatly increased the shelf life for more than 40 days at refrigeration storage and increased the total phenolic content availability.	[63]
Sheep and cow milk	200, and 250 MPa, inlet temperature 85 °C	Treatment at 250 MPa achieved more than 5 log CFU/mL reduction for *B. subtilis* and *B. pumilus* spores.	[64]
Human milk	150 to 300 MPa	Ultra-high-pressure homogenization at 200 MPA attained a lethality > 5 log and did not significantly reduce the immunoglobulin.	[65]
Blueberry juice	200, 250, and 300 MPa, 4 °C and 22 °C, flow rates (0.75, 1.125, and 1.5 L/min)	Treatment at 300 MPa, 4 °C, and 1.5 L/min yielded 54% more anthocyanins. In addition, 22 °C favored ascorbic acid retention. Lower PPO activity was also recorded.	[66]
Pomegranate Juice	50, 100, and 150 MPa	High-pressure homogenization treatment at 150 MPa successfully preserved the total phenolic content and antioxidant capacity (DPPH-ARA, FRAP, ABTS-ARA).	[67]
Peach and carrot juices	25, 50, 100, 150, 200, 250, and 300 MPa	Combined juice treated at 200 MPa retained more concentration of polyphenols and carotenoids.	[68]
Pulsed electric field			
Blood orange juice	Energy density 180 kJ/kg and treatment time of ≤ 3000 µs	Pulsed electric field significantly inactivated the microbes, extended the shelf life (15–20 days), and preserved the bioactive compounds.	[69]
Orange juice	Flow rate 60 mL/min, field strengths 19 kV/cm, 1250 pulses per second, pulse width 2 µs, and total treatment time of 181 µs	Pulsed electric field combined with antimicrobial caps exhibited lowest mold and yest populations. No significant differences in vitamin C and total phenolic compounds after treatments. Stability was maintained for 5 weeks at 10 °C but vitamin C was lost.	[70]
Shalgam juice	Field strength of 1 kV cm^−1^	Pulsed electric field-treated sample exhibited lower lactic acid bacteria and total aerobic mesophilic bacteria after 70 days of storage. Insignificant effect was recorded for antioxidant activity and total phenolic content.	[71]
Pomegranate fermented beverage	Field strength of 11.7 and 18.8 kV/cm, and 15 and 20 µs of pulse-width	A reduced microbial load and slight reduction in bioactive compounds were observed during storage.	[72]
Kiwi-carrot juice	Field strength 35.86 kV/cm for 2400 µs	PME was successfully inactivated, and degradation of ascorbic acid and phenolic compounds was observed.	[73]
Cantaloupe juice	Electric field intensity 15, 20, 25, and 30 kV/cm for 400 µs and treatment times 200, 400, 600 and 800 µs with 20 kV/cm electric field strength	Pulsed electric field combined with natural preservatives improved the inactivation of *S. cerevisiae*, prolonged shelf life, and better preserved vitamin C levels.	[74]
Milk-based beverage	Levels of pulses 20, 50, and 80	Beverage treatment was performed at 80 pulses with no preservative, and stored for 6 days at 5 °C.	[75]
Sohiong juice	Field strength 10 kV/cm for 60 s	Significant increase of 11%, 20%, 12% and 89% was recorded for total anthocyanin content, total phenolic content, DPPH inhibition, and ascorbic acid, respectively.	[76]
Apple juice	9.6 kV/cm field strength, 20 min treatment time, 1000 Hz frequency, and 50% duty ratio	Pulsed electric fields combined with antibacterial agents successfully reduced bacteria and maintained organic acids in apple juice.	[77]
Pulsed magnetic field			
Orange juice	5 to 7 T and 5 to 30 pulses	Pulsed magnetic field reduced the activity of yeast, mesophilic bacteria, and mold. Partially inactivated POD and PME activities and slightly reduced the antioxidant capacity, phenolic compounds, and ascorbic acid.	[78]
Tomato, lettuce, carrot, and cucumber juices	0 to 8 T and 10 to 60 pulses	Pulsed magnetic field indicated superior antibacterial effect against *E. coli* O157:H7 in all the vegetable juices.	[79]
Cloudy apple juice	5 to 7 T and 5 to 30 pulses	PPO, POD, and PME activities were successfully inhibited, and significant decrease in ascorbic acid was observed. No change in DPPH activity and phenols was recorded.	[80]
Orange Juice	2, 4, and 6 T for 5, 10, and 15 min	Successfully reduced the bacterial load, yeast counts, and mold counts at 4 T for 15 min.	[81]
Cucumber, carrot, spinach, and bitter gourd juices	3 times under 8 T and 60 pulses	Pulsed magnetic fields combined with Litseacubeba essential oil completely destroyed the *E. coli* O157:H7.	[82]

**Table 2:** Application of high-pressure carbon dioxide, ultrasound treatment, and radiation processing for shelf life extension and retention of bioactive compounds in liquid foods.

Liquid Food	Processing Conditions	Key Findings	Ref.
High-pressure carbon dioxide			
Sugarcane juice	Pressure 74–351 bar, temperature 33–67 °C, and holding time 30–70 min	Successful reduction was observed for yeast, molds, mesophiles, lactic acid bacteria, PPO, and POD. Supercritical carbon dioxide combined with mild temperature preserved the cane juice.	[83]
Mango in syrup	20 MPa, 60 °C for 30 min	Dense phase carbon dioxide completely inactivated pathogens, PPO, and POD. The shelf life was documented to be less than 86 days, and higher content of total phenols and vitamin C was recorded.	[84]
Pomegranate juice	12.7 MPa, 45 °C, 40 min	Optimal conditions produced a microbiological stable juice up to 28 days of storage at 4 °C. Total phenol content was maintained, while antioxidant activity was decreased during storage.	[85]
Peach, apple, and pear juices	20 MPa, 20, 30, 40, 50, 60 and 70 °C, 20 min	Lower enzymatic activity and slight decline in phenolic compounds were documented.	[86]
Apple juice enriched with Pfaffia glomerata root extract	Pressure 8 and 21 MPa, temperature 40 and 60 °C, CO_2_ volume ratio 20 and 50%	Supercritical carbon dioxide treatment successfully maintained the fructooligosaccharide content.	[87]
Blackcurrant juice	10, 30, and 60 MPa for 10 min at 45 °C	Supercritical carbon dioxide successfully enhanced the stability of total anthocyanins, vitamin C, and antioxidant capacity.	[88]
Ultrasound treatment			
Jabuticaba juice	6.3, 15.9, 25.5, and 36 W/cm^2^	HIUS effectively inactivated PPO, POD, and PME, retained anthocyanin, exhibit phenolic stability, increased gallic acid, and maintained ellagic acid levels.	[89]
Brazil nut beverage enriched with *Opuntia stricta* var. *dillenii* extract	Amplitude 20–80%, 2–12 min	Ultrasounds effectively inactivated microbes and preserved the total phenolic, total flavonoid, and total betaxanthin content.	[90]
Strawberry clear juice	DEUP 20/40 kHz frequency at 60 °C for 5 min (Sequential operation mode), FSDUP 20 + 40 kHz at 60 °C for 5 min (simultaneous operation mode)	DEUP and FSDUP ultrasound treatment increased the storage period up to 14 and 21 days, respectively. DPPH activity, ascorbic acid, anthocyanins, flavonoids, and phenols were reduced during storage.	[91]
Cloudy apple juice	300, 500, and 700 W for 10 min	Ultrasound combined with phloretin resulted in reduction in *Staphylococcus aureus* and *Escherichia coli* and maintained the quality after 14 days of storage.	[92]
Tomato juice	50, 60, and 70 °C for 5, 10, and 15 min	Thermosonication greatly inactivated the microbes and enhanced the content of total phenols, flavonoids, ascorbic acid, and lycopene. Antioxidant capacity was maintained.	[93]
Black carrot juice	0, 4, 8 and 12 min at 24 kHz	Considerably decreased the microbial population and increased the antioxidant properties, ascorbic acid, and phenolic content.	[94]
Spinach juice	200 W, 400 W, and 600 W, 30 kHz, 20 min, 60 °C	Thermosonication significantly reduced the microbial load, and inactivated PPO and POD. Improved the total flavonols, total phenolic, total flavonoid, anthocyanin, chlorophyll, carotenoid, and antioxidant activities (FRAP and DPPH assay).	[95]
Melon juice	Intensities 27 and 52 W/cm^2^, time 10 and 30 min, duty cycle 30 and 75%	Microbial load was greatly reduced, total carotenoids and antioxidant capacities were enhanced, total phenolic content was reduced.	[96]
Kinnow fruit juice	Frequency 40 kHz, power 120 W, time 30–90 min, temperature 30–70 °C	Optimal ultrasound treatment enhanced the total phenolic content, whereas vitamin C and antioxidant activity decreased.	[97]
Clear strawberry juice	60 °C for 5 min, 55 °C for 15 min (DCU), 60 °C for 15 min, 55 °C for 20 min (SDU)	Thermosonication (SDU-60 °C) reduced the microbial load, inactivated the PPO enzyme, and reduced the loss of total phenolic content.	[98]
Blackberry juice	Temperature 64 and 86 °C, amplitude 60% and 90%, and time 114 s and 517 s	Thermosonication (60%, 86 °C) delivered excellent retention of antioxidant activity, phenolic compounds, and anthocyanins.	[99]
Radiation processing			
Coconut water	Excimer lamp 222 nm, UV LED sources at 257, 267, and 286 nm	UV-treated sample achieved 12 days of shelf life. Ascorbic acid and phenolics were decreased with increase in fluence.	[100]
Cold brew coffee	UV wavelength of 254 nm, flux 12 L min^−1^, 18 °C	UV-C extended the shelf life up to 14 days (4 °C) and presented higher content of chlorogenic acid and total polyphenols.	[101]
Coconut water	Dose of 11.52 J/mL, and flow rate of 20.8 mL/s	UV-C radiation combined with nisin produced microbiologically stable coconut water up to 24 days of refrigerated storage. A consistent reduction in total flavonoids, total phenolic compounds, and antioxidant activity was documented.	[102]
Mulberry juice	14 J/cm^2^ for 2, 4, and 8 s	Pulsed light decreased the microbial load and slightly reduced the anthocyanin content at exposure time of 8 s.	[103]
Mulberry juice	Optimized conditions: Exposure length of 6.5 s, light source 10 cm, and sample width 1 mm.	Pulsed light at optimal condition increased the total phenolics and total flavonoids, whereas no difference was observed in total anthocyanin content.	[104]
Grape juice	Fluences of 13, 40, and 66 J/cm^2^ and flow rate 60 mL/min	Pulsed light successfully reduced *E. coli* and slightly affected total phenolic, ascorbic acid, and anthocyanins content.	[105]
Mothers’ milk	5 kGy combined with 4 antimicrobial formulations	γ-irradiation combined with 3 and 4 formulations eliminated all tested pathogens, and immunoglobulins was not altered significantly.	[106]
Broccoli sprout juice	2, 4, 6, 8, 10, and 12 kGy	Sulforaphane content was reduced with the increase in electron beam irradiation dose.	[107]
Goat milk	2, 3, 5, and 7 kGy	Electron beam irradiation (2 kGy) significantly reduced the total microbial count.	[108]

**Table 3:** Application of ozone processing, cold plasma, and membrane processing for shelf life extension and retention of bioactive compounds in liquid foods.

Liquid Food	Processing Conditions	Key Findings	Ref.
Ozone processing			
Pasteurized skim milk	1.5, 5, and 10 ppm	Total viable count was reduced and shelf life was extended up to 15 days of storage.	[117]
Kinnow juice	150 mg/h for 5, 10, and 15 min	Microbiologically stable juice was produced and stored for 3 months. Total flavonoids, phenolics, and antioxidant potential was increased.	[118]
Kinnow juice	150 mg/h for 5, 10, 15 min	Microbial population was reduced and total flavonoids, phenols, antioxidant activity, and DPPH activity were moderately enhanced.	[119]
Watermelon juice	Flow rate of 1 L/min for 5, 10, 15, 20, and 25 min	Microorganism were reduced, PME activity was not altered, and lycopene, ascorbic acid, and total phenolic contents were degraded.	[120]
Mango nectar	40 ppm, 70 °C for 30 min and 20 ppm, 76 °C for 30 min	Microbial load was reduced and highest vitamin C, total carotenoids, and total phenolic values were recorded.	[121]
Sugarcane juice	Flow rate 10 g/h, time 5, 10, 15 min, and temperature 18–20 °C	PPO activity was reduced with longer exposure time, and total phenol and flavonoid contents were reduced.	[122]
Cold plasma			
Kiwifruit juice	Optimized: 30 kV/5 mm/6.7 min Extreme: 30 kV/2 mm/10 min	Shelf life of optimized cold plasma-treated sample was 60, 80, and 100 days at 25 °C, 15 °C, and 5 °C, respectively. More than 50% ascorbic acid loss was documented.	[123]
Pineapple juice	Optimized: 38 kV/631 s Extreme: 45 kV/900 s	Shelf life of optimized cold plasma-treated sample was 25, 50, and 90 days at 25 °C, 15 °C, and 5 °C, respectively. Bioactive substances reduced during storage.	[124]
Pineapple juice	Optimized: 38 kV/631 s Extreme: 45 kV/900 s	Optimized non-thermal plasma extend the shelf life and retained ascorbic acid, total phenol, antioxidant capacity, and flavonoids.	[125]
Dragon fruit juice	Voltage level 10, 20, 30 kV and time 10, 20, 30 min	Atmospheric cold plasma produced microbiologically stable juice for 28 days at 4 °C and increased total phenols. Antioxidant levels and flavonoids were decreased.	[126]
NFC apple juice	Voltage 65 kV, Air 25%, and gas flow rate 470 L/h.	Successfully reduced *E. coli* for 30 days of storage. Ascorbic acid, phenolics, and antioxidant properties were better preserved. Also, chlorogenic acid, l-epicatechin, and cianidanol were preserved.	[127]
Raw buffalo milk	Voltage 70 kV for 15 min	Cold plasma reduced the microbial load, enhanced the microbial stability, and prolonged the shelf life.	[128]
Cloudy apple juice	Gas feed-simulated air (20% oxygen, 80% nitrogen, Gas feed-combined gas (10% oxygen, 90% oxygen), and duration 30 to 150 s	Atmospheric cold plasma reduced *A. acidoterrestris* spores, mold and yeast counts increased during storage, and antioxidant activity remained stable, whereas total phenolic content increased post-storage.	[129]
Coconut milk	Voltage 50 kV, 60 kV, 70 kV, and time 30 s, 60 s, and 90 s	Atmospheric cold plasma successfully reduced the colony count.	[130]
Orange juice	Voltage 20, 25, and 30 kV for 10 min	Enzymes and microbes were successfully inactivated, and ascorbic acid content was degraded.	[131]
Camu-camu juice	Frequency 200, 420, 583, 698 and 960 Hz for 15 min	PPO, POD, and anthocyanins considerably degraded, and concentration of ascorbic acid was increased at higher excitation frequencies.	[132]
Membrane processing			
Watermelon juice	Pore size 0.05 mm, area 4 m^2^, pressure 1 bar, and temperature 37 °C	Successfully reduced the microbial load and comparable shelf life to pasteurized juice (14 days). Effectively concentrated flavonoids, lycopene, and phenolic compounds.	[133]
Cashew apple juice	Molecular weight cut-offs 5, 10, 30, 50 kDa, and pressures 35, 69, 103, and 138 kPa	Ultrafiltered juice was markedly stored for 12 weeks. Tannic acid increased, whereas ascorbic acid and total polyphenol content reduced during storage.	[134]
Sugarcane juice	Molecular weight cut-offs 30 kDA, pressure 104 kPa, and cross flow rate 30 L/h	Clarified juice was stable for 9 weeks (4 °C) with 80% recovery of polyphenols.	[135]
Shalgam Juice	Filter diameter 0.45 µm	Microfiltration reduced the microbial population and increased the shelf life when stored at 4 °C.	[136]
Prickly pear juices (red/green fruit)	Enzyme concentration 4.75 U/mL and 4.65 U/mL; Time 37 min and 35 min; Temperature 55 °C for both fruits	Microfiltration combined with enzymatic treatment yielded microbiologically safe juices for 90 days of storage (4 °C).	[137]
Kombuchas drink	Porosity 10^−7^ to 10^−5^ m, pressure 1 bar, rejection rate 99.99%, filtration surface 0.027 m^2^, and hydraulic permeability 127.20 L h^−1^ m^−2^ bar^−1^	Microfiltration reduced the fermentation microorganism (53.93%).	[138]
Cashew apple juice	Optimal conditions: pore size 0.2 µm and 138 kPa	Microfiltration combined with centrifugation preserved the 87% ascorbic acid content.	[139]

**Table 4:** Application of combined technologies for shelf life extension and retention of bioactive compounds in liquid foods.

Combined Technologies	Liquid Food	Processing Conditions	Key Findings	Ref.
Ultrasonication, Ozonation	Strawberry-cantaloupe functional drink	Power 300 W, frequency 25 kHz, 25 °C, and 10 min; Concentration 300 mg/L, flow rate 1 L/min, 25 °C, and 10 min	Combined treatment extended the shelf life for two months and retained the bioactive compounds (total phenolics, total flavonoids, total carotenes, ascorbic acid, DPPH, ABTS, and FRAP).	[148]
Pulsed electric field, High-power ultrasound	Strawberry juice	30 kV/cm, 100 Hz, 1.5–4.5 min; Amplitude 25%, pulse 50%, 2.5–7.5 min	Combined treatment markedly improved anthocyanin stability during 7 days of storage.	[149]
Ultrafiltration, Ozone processing	Sugarcane juice	30 kDa polysulphone hollow fiber, transmembrane pressure 104 kPa, cross flow rate 30 L/h; Concentration 3 ppm, flow rate 4.6 L/min, time 8.2 min	Combination of ultrafiltration and ozone successfully achieved microbial and enzyme inactivation. Caffeic acid was decreased and vitexin and its derivatives did not reduce significantly.	[150]
Microfiltration, UV-C	Skim milk	Pressure 75 kPa, cross-flow velocity 7 m/s, membrane area 0.312 m^2^, pore size 1.4 µm; UV-C dosage 13.1–39.3 mJ/cm^2^	Combination of microfiltration and UV-C extended the refrigerated shelf life for 40 days. Bioactive serum proteins were not damaged.	[151]
Pulsed electric field, High-power ultrasound	Strawberry juice	30 kV cm^−1^, 100 Hz for 1.5, 3, and 4.5 min; Amplitude 25%, Pulse 50%, for 2.5, 5.0, and 7.5 min.	Combined technologies documented that hydroxycinnamic acids and total phenols were the most stable bioactive compounds. Condensed tannin and flavonol stability depend upon duration of both treatments.	[152]
Ultra-filtration, High-pressure processing	Chestnut rose juice	100, 5, and 5 kDa; 500 MPa for 6 min	Combination of both technologies retained the total flavonoids, but significant reduction in kaempferol, quercetin, catechin, and myricetin was documented.	[153]
Pulsed electric field, Ultrasonication	Spinach juice	Pulse frequency 1 kHz, electric field strength 9 kV/cm, temperature 30 °C, flow rate 60 mL/min, and time 335 µs; Frequency 40 kHz, temperature 30 °C, time 21 min, and radiating power 200 W	Inactivation of PPO and POD was enhanced, and highest value of vitamin C, carotenoids, flavonols, anthocyanins, flavonoids, antioxidant capacity, DPPH, phenolic, and total chlorophyll was achieved with combined technologies.	[154]
Ultra-filter membrane, Pulsed electric field	Sugarcane juice	Pressure 1 bar, and pore size 10 kDa; Field strength 20, 30, and 40 kV/cm, pulse width 100, 150, and 200 µs	Reduction in PPO and POD activity and retention of ascorbic acid content were documented.	[155]
High-pressure processing, Pulsed electric field	Concord grape juice	600 MPa, 5 °C, and 3 min; 0.85 kV/cm and 300 pulses	Combined treatment reduced the microbial load, enhanced the shelf life, and increased the antioxidant activity and antioxidant contents.	[156]
Microfiltration, Ultrasonication	Skim milk	Pore size 1.4 µm, length 500 mm, area 0.312 m^2^, pressure 75 kPa, and cross flow velocity 7.0 m/s; Output power 720 W, pulse time 5 s, intermittent time 5 s, and total ultrasonic time 3, 9, or 15 min	Combination of the two techniques removed the bacteria, extended the shelf life for at least 40 days, and retained the bioactive proteins (IgG, IgA, and IgM).	[157]
Microfiltration, High-pressure processing	whole milk	Pore size 1.4 µm; 600 MPa for 5 min	Combined treatment reduced the total bacterial and *E. coli* count to 4 log units.	[158]
Ultrafiltration, Ozone treatment	Sugarcane juice	Hollow fiber membranes 30 kDa (TMP of 104 kPa and CFR of 30 L/h); Ozone concentration 3.12 ppm, flow rate 4.58 L/min, and time 8.2 min.	Hurdle technology reduced the spoilage rate and phenolic content degradation.	[159]

## Data Availability

The original contributions presented in the study are included in the article, further inquiries can be directed to the corresponding author.
